# A Case of Pediatric Posterior Reversible Encephalopathy Syndrome (PRES) Secondary to Post-streptococcal Glomerulonephritis: A Literature Review and Assessment of Treatment Modalities

**DOI:** 10.7759/cureus.25113

**Published:** 2022-05-18

**Authors:** Abdurrahman F Kharbat, Pedro Calles, Allison Ogle, Tetyana L Vasylyeva, Kerrie Pinkney

**Affiliations:** 1 Neurological Surgery, Texas Tech University Health Sciences Center, Amarillo, USA; 2 Family Medicine, Texas Tech University Health Sciences Center, Amarillo, USA; 3 Pediatrics, Texas Tech University Health Sciences Center, Amarillo, USA

**Keywords:** radiological findings in pres, atypical pres, pediatric hypertensive emergency, posterior reversible encephalopathy syndrome (pres), post-streptococcal glomerulonephritis

## Abstract

Posterior reversible encephalopathy syndrome (PRES) is a disorder that most commonly affects adults, and is characterized by neurologic symptoms such as encephalopathy, seizures, headaches, and visual disturbances. It usually occurs in the context of other systemic disturbances that result in hypertensive crises, such as renal failure, cytotoxic drugs, and autoimmune conditions. In children, it rarely manifests following chemotherapy induction or hematopoietic stem cell transplantation. No cases have been reported in the English literature connecting renal dysfunction and hypertensive emergency secondary to post-streptococcal glomerulonephritis (PSGN) with PRES. We present a case of an eight-year-old boy, who developed a constellation of symptoms suggestive of PSGN and later developed PRES. PRES is often confirmed upon suspicion through brain MRI showing subcortical edema of various brain regions including occipital, temporal, or parietal cortices. Our patient demonstrated subcortical edema of the bilateral occipital lobes and right cerebellar hemisphere, with positive antistreptolysin O (ASO) titers demonstrating PSGN as the likely etiology for his hypertensive emergency. Management included antihypertensive and anticonvulsant treatment, which allowed the resolution of the offending hypertensive emergency that resulted in PRES. Our case adds to the growing body of literature on PRES and describes a new etiology of pediatric PRES secondary to PSGN.

## Introduction

Posterior reversible encephalopathy syndrome (PRES) is a syndrome characterized by reversible subcortical vasogenic edema in the brain that manifests in patients with acute neurological symptoms (i.e., seizures, encephalopathy, headache, and visual disturbances) [1-6]. It has been reported to occur in the setting of various systemic illnesses, such as renal failure, dramatic blood pressure fluctuations, cytotoxic drugs, autoimmune disorders, and preeclampsia or eclampsia [2,4,6]. Pediatric PRES may seldom occur and often arises in the setting of chemotherapy initiation for acute lymphocytic leukemia (ALL), which results in endothelial cytotoxic injury [4]. Even more rarely, pediatric PRES may develop following hematopoietic stem cell transplantation (HSCT) and during prophylactic cranial irradiation [4].

Although the pathogenesis is under debate, the leading theory purports that PRES is due to disruption in the autoregulation of cerebral perfusion, which subsequently results in the breakdown of the blood-brain barrier [1-10]. This allows the extravasation of plasma and macromolecules into the brain interstitial space [4,6]. The posterior brain is particularly vulnerable to this phenomenon due to relative decreases in sympathetic innervation, which results in decreased propensity to autoregulation of blood pressure [6]. This theory prevails due to the resolution of PRES symptoms clinically and radiologically following treatment of acute hypertension [6].

Clinical signs of PRES vary and are limited by a small sample of reported cases in the literature, but common symptoms include focal neurologic deficits or signs and symptoms of increased intracranial hypertension, including headache, nausea, vomiting, diplopia, and visual field abnormalities [4]. Encephalopathy (ranging from mild confusion to deep stupor) is common, and generalized tonic-clonic seizures occur in 60-75% of patients [5-9]. What is more worrisome, albeit less common, is status epilepticus, in which PRES can be the suspected etiology when bilateral occipital sharp waves are present on EEG [5-7].

Brain imaging via T2 fluid-attenuated inversion recovery sequences may demonstrate predominantly subcortical abnormal T2 signal bilaterally in the cerebellar hemispheres, watershed regions, posterior parieto-temporal regions, and occipital regions [10]. Imaging is often what clues clinicians in on the diagnosis of PRES and is also used to verify the resolution of this syndrome in the setting of clinical improvement [10].

## Case presentation

An eight-year-old previously healthy male was presented to the emergency department (ED) with new-onset seizures. The patient fell and hit his head at school without any loss of consciousness (LOC). He began having severe intractable headaches, blurry vision, fever, vomiting, and nausea three days prior to arrival. In the ED, the patient had altered mental status (AMS) with blood pressure (BP) ranging from 192-155/132-114 mmHg, followed by a witnessed focal seizure with left downward gaze. He received two doses of midazolam with loading dose of Keppra. A non-contrast head CT done in the ED showed no acute changes.

Patient soon became tachypneic and hypoxic, which was thought to be secondary to obstructive sleep apnea due to his large body habitus (>99th percentile). Continuous positive airway pressure (CPAP) was started. Patient had a tonic-clonic seizure the following morning lasting about 10 minutes. He received two 4 mg doses of lorazepam and then a 1000 mg dose of levetiracetam. Patient was then transferred to the pediatric intensive care unit. Due to continued seizures, levetiracetam was scheduled every 12 hours. Upon further questioning, the patient’s parents reported a history of upper respiratory infection approximately three weeks ago, followed by dark urine 1.5 days prior to arrival to the ED. Diagnostic labs and imaging ordered overnight began to result, with resulting studies and values seen in Tables 1, 2.

**Table 1:** Laboratorial analysis demonstrating various abnormalities supporting the diagnosis of PSGN. H: high; WNL: within normal limits; L: low; BNP: brain natriuretic peptide; Ab: antibody; ANA: antinuclear antibody; ASO: antistreptolysin O; CK: creatine kinase; UA: urinary analysis; PSGN: post-streptococcal glomerulonephritis

Study	Value	Normal reference range and units
BNP	980 (H)	<100 pg/mL
Thyroperoxidase Ab	0.70 (WNL)	<9 IU/mL
Normetanephrine, random	180 (WNL)	75 to 375 mcg
Metanephrine, random	78 (L)	140 to 785 mcg
Renin	0.12 (L)	0.25 to 5.82 ng/mL/hr
ANA	0.3 (WNL)	>0.00625 ratio
C3	25 (L)	88 to 201 mg/dL
ASO titer	797 (H)	<276 IU/mL
Blood, UA	3+	None
CK	62 (WNL)	22 to 198 IU/L
Urine myoglobin	306 (H)	0 to 85 ng/mL
Urine creatinine	158.40	Variable
Urine sodium	169	Variable
Urine potassium	90	Variable
Urine protein	1394	Variable
Urine drug screen	Negative	Negative

**Table 2:** Imaging and diagnostic studies demonstrating various indications supporting the diagnosis of PRES and ruling out other pathologies. EEG: electroencephalogram; CXR: chest x-ray; US: ultrasound; TTE: transthoracic echocardiogram; MRI: magnetic resonance imaging; BLL: bilateral lower lobes; WNL: within normal limits; PRES: posterior reversible encephalopathy syndrome

Study	Findings
EEG	Abnormal study, suggestive of posteriorly maximal diffuse neuronal dysfunction and encephalopathy.
CXR	BLL infiltrates that could be pulmonary edema or infiltrates or both.
Renal US	WNL
Renal US with duplex	WNL
TTE	WNL
MRI brain	Large regions of abnormal signal in both cerebral hemispheres, predominantly posteriorly, and in the right cerebellar hemisphere. This appearance is suggestive of posterior reversible encephalopathy syndrome (PRES).

**Figure 1:**
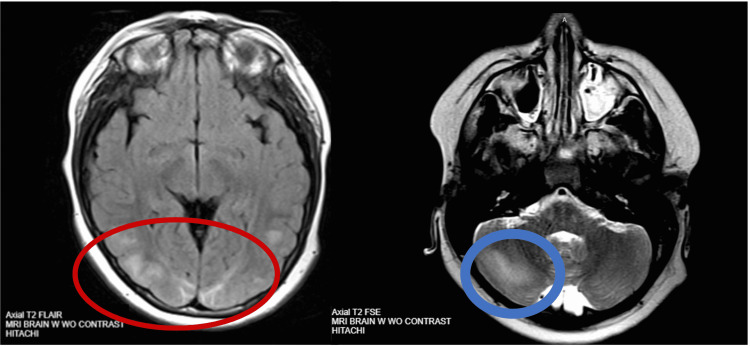
Brain T2 magnetic resonance imaging without contrast. Large regions of abnormal signal in both cerebral hemispheres, predominantly posteriorly (red circle) and in the right cerebellar hemisphere (blue circle), suggestive of PRES. FSE: fast spin echo; FLAIR: fluid-attenuated inversion recovery; PRES: posterior reversible encephalopathy syndrome

Following the results of our workup and further history from the parents, post-streptococcal glomerulonephritis (PSGN), confirmed by elevated antistreptolysin O (ASO) titers, and severe hypertensive emergency resulting in PRES were high on the differential. The diagnosis of PRES was confirmed via axial T2 MRI without contrast demonstrating large regions of abnormal signal in both cerebral hemispheres, predominantly posteriorly in the occipito-parietal lobes, and in the right cerebellar hemisphere (Figure 1).

Amlodipine 5 mg daily and IV furosemide 20 mg were initiated along with azithromycin and ceftriaxone. Once mycoplasma IgM antibody immunoassay was determined to be negative, azithromycin was discontinued. Ophthalmology and nephrology were consulted. Blood pressure continued to be labile and had significant elevations. Labetalol was added as needed for occasional spikes. Amlodipine was increased to 10 mg for further control of blood pressure (BP). Patient’s clinical encephalopathy then resolved. Blood pressure remained unstable, and labetalol was increased to twice daily. Patient continued to have high BP and was on the highest possible dose of amlodipine and on a high dose of labetalol. The decision to start nicardipine drip was then made.

Respiratory failure resolved without continued hypoxia and CPAP was discontinued. BP was less labile and trending downward. Patient was then weaned off nicardipine drip. Labetalol 200 mg BID and amlodipine were continued. Enalapril 2.5 mg was added, due to proteinuria and unstable renal function. Enalapril was increased to 10 mg daily. Blood pressure was controlled with oral medications. Throughout the hospital course, three antihypertensive agents were carefully titrated to slowly reduce BP on average 25% daily to prevent further neurological injury. On hospital day six, the patient was discharged, with a stable renal function and a BP within normal limits. On discharge, the patient was counseled to maintain a sodium-restricted diet.

## Discussion

Given the irreversible sequelae of uncontrolled encephalopathy in any individual and especially in children, the early diagnosis and appropriate treatment of PRES are of key importance in the pediatric population. Brain MRI imaging is at the forefront of the diagnostic workup and should be considered in all children with concerns of encephalopathy and a sterile cerebrospinal fluid analysis excluding pathogenic encephalitis/meningitis.

The treatment of PRES is supportive and consists of resolving the underlying etiology, which is often related to intracranial hypertension, disruption of the blood-brain barrier, or cytotoxic endothelial injury in the brain [1,4,6]. Thus, treatment often consists of initiation of antihypertensives, anticonvulsants, and withdrawal of the offending agents, such as in the setting of chemotherapy induction [4].

Although the withdrawal of chemotherapy is a tough clinical decision to make, Norman et al. presented a case series of three patients with PRES following chemotherapy induction, and the withdrawal of chemotherapy in conjunction with antihypertensives and anticonvulsants proved to be effective in the treatment of these patients [11]. With respect to the anticonvulsants to be used, Morris et al. recommend three to 12 months of seizure prophylaxis, with increased durations in patients who demonstrate EEG changes or recurrent seizures [12]. Studies show that valproic acid and clonazepam are preferable to phenytoin and phenobarbital, or phenytoin, given that the aforementioned drugs do not induce the cytochrome P450 system [13]. Moreover, magnesium has been postulated to aid in the reduction of vasoconstriction in PRES, given its mechanism of competing with calcium at the cellular level and the current study of its prevention of vasospasm following subarachnoid hemorrhage [14].

Our unique case adds to a growing body of literature on the rare constellation of symptoms recognized as PRES. Moreover, pediatric PRES is associated with chemotherapy induction and hematopoietic stem cell transplantation, with no cases ever reported in the English literature establishing PSGN as a cause for renal dysfunction that results in PRES.

## Conclusions

We present the first case of PSGN-associated PRES in English literature. This case highlights some key considerations to deliberate in the care of patients presenting with PRES. The importance of history taking within a workup is made evident throughout this case. The initial workup was focused on neurological symptoms, as they could have related to primary epilepsy or post-concussive syndrome, since the history of recent illness had not been made clear. This history could have helped direct the workup more quickly towards the appropriate diagnosis of PRES secondary to renal dysfunction due to PSGN, and allowed for more efficient management of the patient. Specifically, when considering falls and seizures, it is important to always consider causation. In this case, the patient experienced an unwitnessed fall which led the initial workup team to consider the fall as a cause of the seizures. However, we now know it was more likely that an initial seizure caused that fall. Considering every scenario when starting a patient workup can increase quality of patient care.
